# Are people noticing excessive mistrust in others and how do they understand it? A survey of a UK representative adult population

**DOI:** 10.1017/S0033291725102365

**Published:** 2025-11-03

**Authors:** Ghizlane Slaoui, Helen Beckwith, Sinéad Lambe, Thomas Kabir, Glory Sokunle, Memoona Ahmed, Daniel Freeman

**Affiliations:** 1 Department of Experimental Psychology, University of Oxford, Oxford, UK; 2 Faculté de Psychologie, Université de Strasbourg, Strasbourg, France; 3 Oxford Health NHS Foundation Trust, Oxford, UK; 4 Department of Psychiatry, University of Oxford, Oxford, UK

**Keywords:** adult population, conspiracy beliefs, mistrust, paranoia

## Abstract

**Background:**

If excessive mistrust – for example, holding conspiracy beliefs or experiencing paranoia – is widespread then people should notice it in others. We aimed to assess the degree to which the general population had observed excessive mistrust. Paranoia is a particular form of excessive mistrust. How people understand paranoia, and therefore react to it, could affect its persistence. We also aimed to learn how the general population views paranoia.

**Methods:**

A non-probability online survey was conducted in May 2024 with 1,036 UK adults, quota sampled to match the population for age, gender, ethnicity, income, and region. Nine examples of excessive mistrust in others were presented. Knowledge of paranoia was also assessed.

**Results:**

Participants (*n* = 698, 67.4%) had most commonly encountered a person ‘Saying that they would not get a COVID-19 vaccination because of concerns about the real motivation behind the vaccine rollout’. Least commonly encountered by participants (*n* = 328, 31.7%) was a person ‘Thinking that others are targeting them in order to bully or exploit them, and so isolating from the world and refusing to leave their home’. A total of 854 (82.4%) participants had observed at least one form of excessive mistrust in the past year, most frequently in friends. More mistrustful participants were more likely to observe mistrustful behaviors. Participants endorsed multiple causes of paranoia, with the most endorsed causes being worry and illicit drugs.

**Conclusions:**

The large majority of people have encountered others, primarily individuals they know, exhibiting excessive mistrust. Public understanding of paranoia varies greatly, with diverse definitions and perceived causes.

## Introduction

In the weeks after the start of the COVID-19 pandemic, we presented data in this journal that conspiracy beliefs may have become mainstream (Freeman, Waite et al., 2022). Subsequent evidence is broadly consistent with such a view (Fotakis & Simou, 2023). Conspiracy beliefs are one form of excessive mistrust. A related, but distinct (Alsuhibani et al., 2022; Martinez et al., 2022), type of excessive mistrust is paranoia: thinking that others are targeting you when they are not (Freeman & Garety, 2000). Persecutory ideation is common in the general population (e.g. Bebbington et al., 2013; Bird et al., 2019; Neidhart, Mohnke, Vogel, & Walter, 2024). A quarter of the UK population describes themselves as mistrustful of other people (Freeman & Loe, 2023). Another distinct form of mistrust may be jealousy in relationships, which may range too in severity from normal and understandable to excessive (e.g. Kupfer et al., 2022; Lima et al., 2017). There are of course real dangers that mean mistrust is not always misplaced; it can be absolutely justified and right to be wary. There is however an appropriate balance to be made between trust and mistrust, that will vary by person, place, time, and circumstance. But excessive mistrust, when the fear has become clearly inaccurate and disproportionate, can lead to negative behavioral consequences, such as not taking up suitable medical procedures to the detriment of health (Bierwiaczonek, Gundersen, & Kunst, 2022; Coelho, Foster, Nedri, & Marques, 2022; Freeman, Loe et al., 2022), the breakdown of relationships, and isolation. If excessive mistrust is widespread, then extreme examples should be noticed in the people around us. We report here the first survey on this prediction.

Paranoia is the form of excessive mistrust most commonly studied, understood, and treated within a mental health context (Freeman, Isham, & Waite, 2025). Its most common clinical presentation is persecutory delusions in the context of severe mental health conditions such as psychosis. Though it has a precise meaning in clinical settings, the term is used much more widely in the general population. Common uses highlighted by psychoeducational websites are: ‘Some people might describe a paranoid thought as a type of anxious thought’ (Mind, 2024). We were interested to find out the degree to which the general public’s definition of paranoia aligns with that of clinical researchers. Mental health literacy positively influences attitudes towards mental health help-seeking (Jung, von Sternberg, & Davis, 2017). Furthermore, it is plausible that how the general public perceives the behaviors of people with severe paranoia may impact outcomes. Negative responses may worsen the interactions of the mistrustful person and entrench the fears further; positive responses may increase the likelihood that trust can re-emerge and the paranoia lessen. These responses are likely to be partly determined by levels of knowledge about paranoia. Understanding in which areas public knowledge about paranoia is limited could inform future psychoeducational interventions to the benefit of patients and carers.

Paranoia has increasingly become a topic of study in its own right, with advances in understanding and treatment. Paranoia typically develops in adolescence or young adulthood (Bird, Freeman, & Waite, 2022), and decreases over time (Della Libera et al., 2020; Greenburgh & Raihani, 2022; Saarinen, Granö, & Lehtimäki, 2021), with a slight increase in older age (Östling, 2004). A range of social, cognitive, environmental, and biological processes have been linked to paranoia. Paranoia has been associated with anxiety, worry, and depression (Freeman et al., 2012), as well as to social stress (Veling et al., 2016). In a recent survey of the adult general population, two-thirds of the variance in paranoia was explained by a model containing a range of cognitive and social processes such as within-situation defense behaviors, negative images, negative self-beliefs, discrimination, dissociation, aberrant salience, agoraphobic distress, decreased social support, agoraphobic avoidance, decreased analytical reasoning, and alcohol use (Freeman & Loe, 2023). Heritability of paranoia has been estimated at 50% (Zavos et al., 2014), and a range of genes may be associated with paranoia (Crespi, Read, Salminen, & Hurd, 2018; Sieradzka et al., 2015). Taken together, the evidence indicates that the cause of paranoia is multi-factorial (Freeman et al., 2025). For severe clinical presentations, new psychological treatments have been developed from this perspective that have shown high efficacy (Freeman et al., 2021a), while antipsychotic medications can also clearly reduce paranoia (Leucht et al., 2017).

The overall aim of the present study was to describe for the first time the experiences and understanding of the general public regarding excessive mistrust occurring in other people, with a particular focus on paranoia. We set out to answer three specific questions: Do people observe others being excessively mistrustful? What do people mean when they use the term paranoia? How does the general public understand the particular form of mistrust that is paranoia (inaccurate fears that others intend harm)?

## Methods

### Participants

An online survey was conducted with a quota-sampled UK participant group of 1,128 adults (aged 18 years old or over), representative of the overall UK population for age, gender, ethnicity, income, and region. Participant recruitment was conducted from the 20th of May 2024 to the 29th of May 2024 via Cint, a market research company. Respondents were recruited via a variety of methods: ads and promotions on digital networks, word of mouth, social networks, affiliate marketing, banner ads, online and mobile games, and offline recruitment with mail campaigns. All participants recruited through Cint had opted in to being panel members for the market research company. The study was approved by the Medical Sciences Interdivisional Research Ethics Committee at the University of Oxford (reference R93164/RE001). The survey questions were not accessible until informed consent was given. The survey was completed online via Qualtrics, either on a computer or mobile browser, and took on average 24 minutes to complete.

Sixteen people were excluded for completing the survey in less than one-third of the median completion time; 36 people were excluded for providing answers that were not serious (e.g. nonsense words) to the open-ended questions; six people were excluded for selecting the same answer for every item on at least three different sections; and 34 people were excluded for both non-serious responses to the open-ended questions and selecting the same answer repeatedly across sections. This resulted in a final sample of 1,036 people.

### Assessments

#### Demographics

In the first section of the survey participants indicated their age, gender, level of education, ethnicity, socioeconomic status, marital status, employment status, and religious or spiritual affiliation. These factors have been associated with community attitudes towards individuals with mental illness (Angermeyer & Matschinger, 1997; Bhugra, 1989; Lam, Chan, & Chen, 1996).

#### Experiences of excessive mistrust in other people

In the second section of the survey participants reported any experiences of other people being excessively mistrustful. For nine different examples of extreme mistrust (e.g. ‘Saying that the COVID-19 virus is not real and that the pandemic was a hoax,” ‘Thinking that others are targeting them in order to bully or exploit them, and so isolating from the world and refusing to leave their home.’), they were asked whether they had interacted with anyone in the past year who had shown such behavior. They could respond: No one, One or two people, Quite a few people, or Most people. If they had noticed such behavior in others they were then asked about the nature of their relationship with the mistrustful person (e.g., friend, acquaintance, partner).

#### Understanding paranoia

In the third section of the survey participants were asked about their conceptions and understanding of paranoia. They were first asked an open-ended question: ‘How would you define paranoia, i.e., what do you think it is?’. Participants gave a free text response for their definition of the word paranoia. Participants were then given the following definition based on Freeman and Garety’s (2000) definition of persecutory delusions:

We define paranoia as having an **enduring belief that other people (or organisations) are deliberately trying to harm you when they are not**. It is excessive mistrust of other people. People may think others are trying to harm them socially, physically, or financially when they are not.

Based on this definition participants were asked if they knew anyone who experiences paranoid thoughts (answer choices: ‘No, I do not know anyone who has these thoughts’; ‘Yes, I know someone who previously had these thoughts’; ‘Yes, I know someone who currently has these thoughts’), and, if so, what their relationship was to that person (‘Partner’; ‘Someone at work’; ‘Close friend’; ‘Friend’; ‘Family member’; ‘Acquaintance’; ‘Stranger’), and how they realized this person was experiencing paranoid thoughts (‘They told me’; ‘I noticed it in their behavior or something they said’; ‘Someone else pointed it out to me’). Participants were then asked to rate their level of understanding of paranoia (‘I do not understand it at all’; ‘I understand a little why people may get paranoid’; ‘I have some understanding of why people get paranoid,” ‘It is very understandable why people get paranoid’).

Participants were asked about causes of paranoia. They were asked to indicate on a 5-point scale (*Strongly disagree*, *Disagree*, *Neither agree nor disagree*, *Agree*, *Strongly agree*) to what extent they believed each of 34 different factors play a role in causing paranoia (e.g., ‘chemical imbalance in the brain’, ‘Social isolation’). Participants were also asked to indicate what they believed to be the main cause of paranoia out of the items presented. The items were derived from Part C of the Trauma-Voice Association Questionnaire (van den Berg, Hardy, & Staring, 2015), a local NHS service intake questionnaire, Freeman’s (2016) model of persecutory delusions, and from discussions within the authorship team (which included a lived experience researcher).

Participants were then asked questions about their ideas concerning the onset, timeline, and potential gender differences in paranoia.

Finally, participants were asked to indicate on a 5-point scale (*Strongly disagree*, *Disagree*, *Neither agree nor disagree*, *Agree*, *Strongly agree*) to what extent they believed each of 13 approaches could help someone with paranoia (e.g. ‘Getting more sleep’, ‘Seeing a therapist’).

#### Revised Green et al. paranoid thoughts scale (R-GPTS; Freeman, Loe et al., 2021 ***)***

The R-GPTS was used to measure the participants’ own levels of paranoia. This scale comprises eight items concerning ideas of reference (Part A; e.g. ‘I spent time thinking about friends gossiping about me’) and 10 items concerning ideas of persecution (Part B; e.g. ‘People have been hostile towards me on purpose’). Each item is rated for the past month on a 5-point scale (from 0 = *not at all* to 4 = *totally*). Higher scores on each scale indicate higher levels of paranoia.

#### Generic conspiracist beliefs (GCB) scale (Brotherton, French, & Pickering, 2013)

The GCB was used to assess participants’ own levels of conspiracy belief endorsement. This is a 15-item scale where each item is rated on a 5-point Likert scale (1 = *definitely not true*; 2 = *probably not true*; 3 = *not sure/cannot decide*; 4 = *probably true*; 5 = *definitely true*). Example items include: ‘The spread of certain viruses and/or diseases is the result of the deliberate, concealed efforts of some organisation’, or ‘A lot of important information is deliberately concealed from the public out of self-interest’. A higher score indicates greater levels of conspiracy-type assumptions and beliefs.

### Analysis

Statistical analyses were conducted using SPSS version 29 (IBM Corp., 2023) and R version 4.2.1. (R Core Team, 2021). The analysis was largely descriptive in nature, showing frequency responses for items. Due to the ordinal nature of the data, polychoric correlation analyses were carried out between the nine mistrust examples. A summed total score was also calculated for the mistrust examples, which was then correlated with paranoia and conspiracy beliefs scores.

A content analysis was conducted on participants’ free text responses to the question: ‘How would you define paranoia, i.e., what do you think it is?’ Participants’ answers were coded by a researcher (GS). The following steps were undertaken in conducting the content analysis: familiarisation with the data, initial coding, grouping codes into themes, refining themes through discussion sessions with the research group, and quantifying the themes by counting how many participant answers fell into each theme. Each response was only assigned one code. A code hierarchy was built based on the closeness of the definition to our definition of paranoia. Coding a definition into the ‘Having ideas that others are trying to harm them’ code always took priority over other codes. Inter-rater reliability was assessed by selecting 100 definitions at random, which were independently coded by two raters, GS and MA (research assistants), using the list of codes and the code hierarchy. Cohen’s Kappa coefficient was calculated to quantify the inter-rater reliability of this coding.

An exploratory factor analysis (EFA) was conducted using R version 4.2.1. (R Core Team, 2021) to identify the underlying structure of causes for paranoia endorsed by the sample. A confirmatory factor analysis (CFA) was then conducted to confirm the structure identified by the EFA. The total sample (*n* = 1036) was first randomly split into two subsamples: a derivation sample (*n* = 725) and a confirmatory sample (*n* = 311). The EFA was conducted on the derivation sample (*n* = 725). Prior to EFA, we employed the Kaiser–Meyer–Olkin measure of sampling adequacy and Bartlett’s test for sphericity to assess the suitability of factor recovery using the observed dataset. Parallel analysis was used as the criterion for factor retention for subsequent rotation using the oblimin algorithm. Items with low communalities (<0.30), items that had low loadings on all factors (<0.30), and items that loaded onto multiple factors were excluded from the final model. Seven factors were retained. Subsequently, a CFA was conducted to test the seven-factor structure identified by the EFA. The maximum likelihood estimation method was applied to both the derivation sample (*n* = 725) and confirmatory sample (*n* = 311). Model fit was evaluated using a Comparative Fit Index (CFI) and Tucker–Lewis index (TLI) of >0.95, a Root Mean Square Error of Approximation (RMSEA) of <0.06, and a Standardized Root Mean Square Residual (SRMR) of <0.08 (Hu & Bentler, 1999).

Exploratory analyses were conducted to examine whether paranoia is perceived and understood differently across demographic groups (see Supplementary materials B). Perceived causes of paranoia were grouped according to the factor structure identified in the EFA, with factor scores computed by summing the scores of the items that constituted each factor. To compare factor scores across male and female genders, independent sample *t*-tests were conducted. Multivariate Analyses of Variance (MANOVA) were conducted to compare factor scores across age, ethnicity, and level of education. Following a significant MANOVA, one-way ANOVAs were conducted to identify where group differences occurred, followed by post-hoc comparisons. A full description of the analysis can be found in Supplementary materials B: Analysis of demographic factors.

## Results

### Participant group

A summary of the socio-demographic information of the final participant group (*N* = 1,036) can be seen in Table 1. The mean age of the participant group was 48.2 (SD = 16.8) years old. The mean R-GPTS Part A score was 7.3 (SD = 8.1) and Part B score was 7.5 (SD = 10.3). These R-GPTS scores are a little lower than the UK representative group of 10,382 reported by Freeman and Loe (2023) (mean R-GPTS Part A score = 9.4 (SD = 9.1) and Part B score = 9.7 (SD = 11.5). The mean GCB score was 35.9 (SD = 14.6).

**Table 1.** Socio-demographic information about the participant group

Demographic characteristics	*n*	%
**Gender**		
Male	507	48.9%
Female	523	50.5%
Non-binary/other gender	5	0.5%
Prefer to self-describe	1	0.1%
**Marital status**		
Single	307	29.6%
Cohabiting	145	14.0%
Married	422	40.7%
Civil Partnership	24	2.3%
Divorced	74	7.1%
Separated	11	1.1%
Widowed	45	4.3%
Prefer not to say	8	0.8%
**Level of education**		
No qualifications	28	2.7%
Primary	5	0.5%
Secondary	290	28.0%
Further	282	27.2%
Certificate of higher education	304	29.3%
Post-graduate qualifications	127	12.3%
**Employment status**		
Unemployed	53	5.1%
Employed full-time	479	46.2%
Employed part-time	130	12.5%
Self-employed	60	5.8%
Retired	181	17.5%
Student	35	3.4%
Homemaker	51	4.9%
Voluntary	4	0.4%
Disabled/long-term sick leave	43	4.2%
**Annual household income**		
<£15,000	139	13.4%
£15,000 – £19,999	91	8.8%
£20,000 – £29,999	211	20.4%
£30,000 – £39,999	174	16.8%
£40,000 – £49,999	144	13.9%
£50,000 – £59,999	77	7.4%
£60,000 – £69,999	57	5.5%
£70,000 – £99,999	61	5.9%
£100,000+	40	3.9%
Prefer not to say	42	4.1%
**Religion/spirituality**		
Atheism	402	38.8%
Agnosticism	45	4.3%
Buddhism	6	0.6%
Christianity	481	46.4%
Islam	39	3.8%
Judaism	5	0.5%
Hinduism	12	1.2%
Sikhism	1	0.1%
Jainism	1	0.1%
Spiritual	17	1.6%
Other	27	2.6%
**Ethnicity**		
**White**		
British/English/Northern Irish/Scottish/Welsh	846	81.7%
Irish	43	1.3%
Any other White background	32	3.1%
**Mixed/multiple ethnic groups**		
White and Black Caribbean	12	1.2%
White and Black African	10	1.0%
White and Asian	11	1.1%
Any other Mixed/Multiple ethnic background	3	0.3%
**Asian/Asian British**		
Indian	13	1.3%
Pakistani	20	1.9%
Bangladeshi	2	0.2%
Chinese	10	1.0%
Any other Asian background	4	0.4%
**Black/African/Caribbean/Black British**		
African	35	3.4%
Caribbean	7	0.7%
Any other Black/African/Caribbean background	2	0.2%
**Other ethnic group**		
Arab	4	0.4%
Any other ethnic group	1	0.1%
Prefer not to say	11	1.1%

### Experiences of excessive mistrust in others

**Figure 1.**
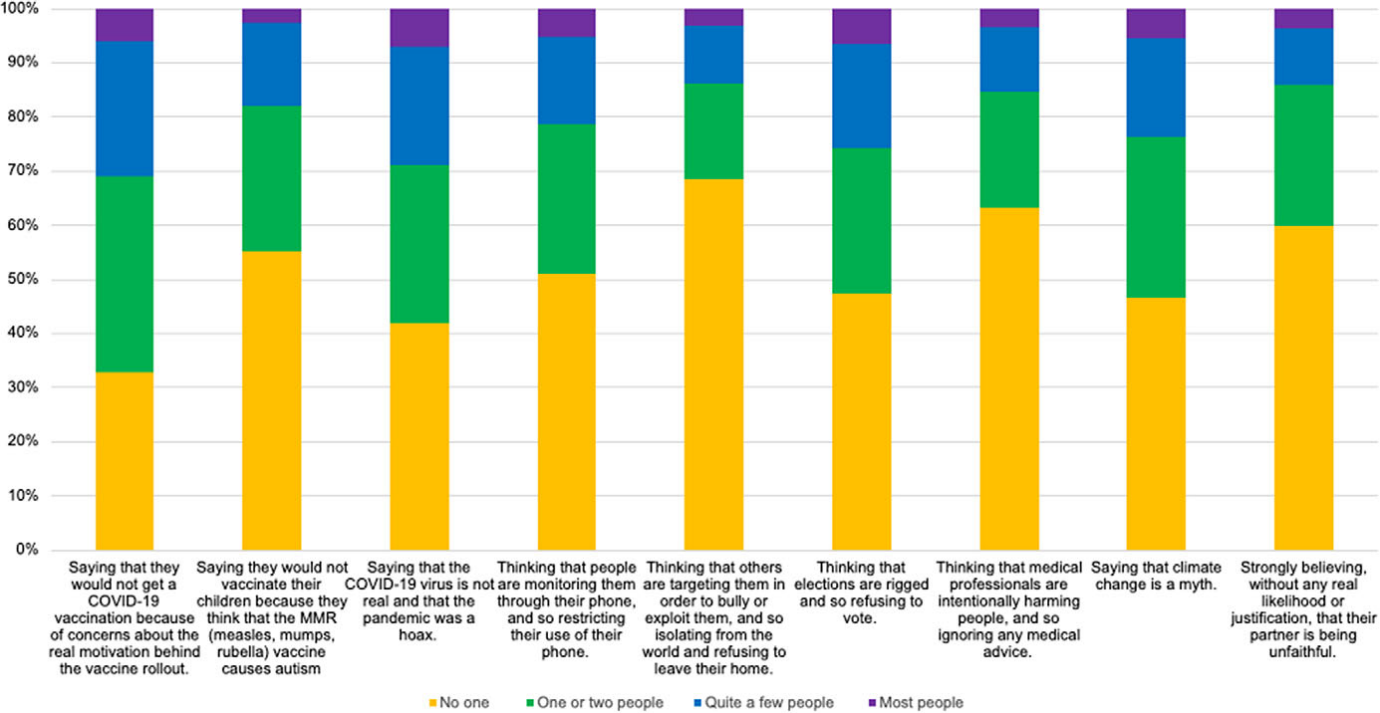
Observing mistrustful behaviors in other people.

**Figure 2.**
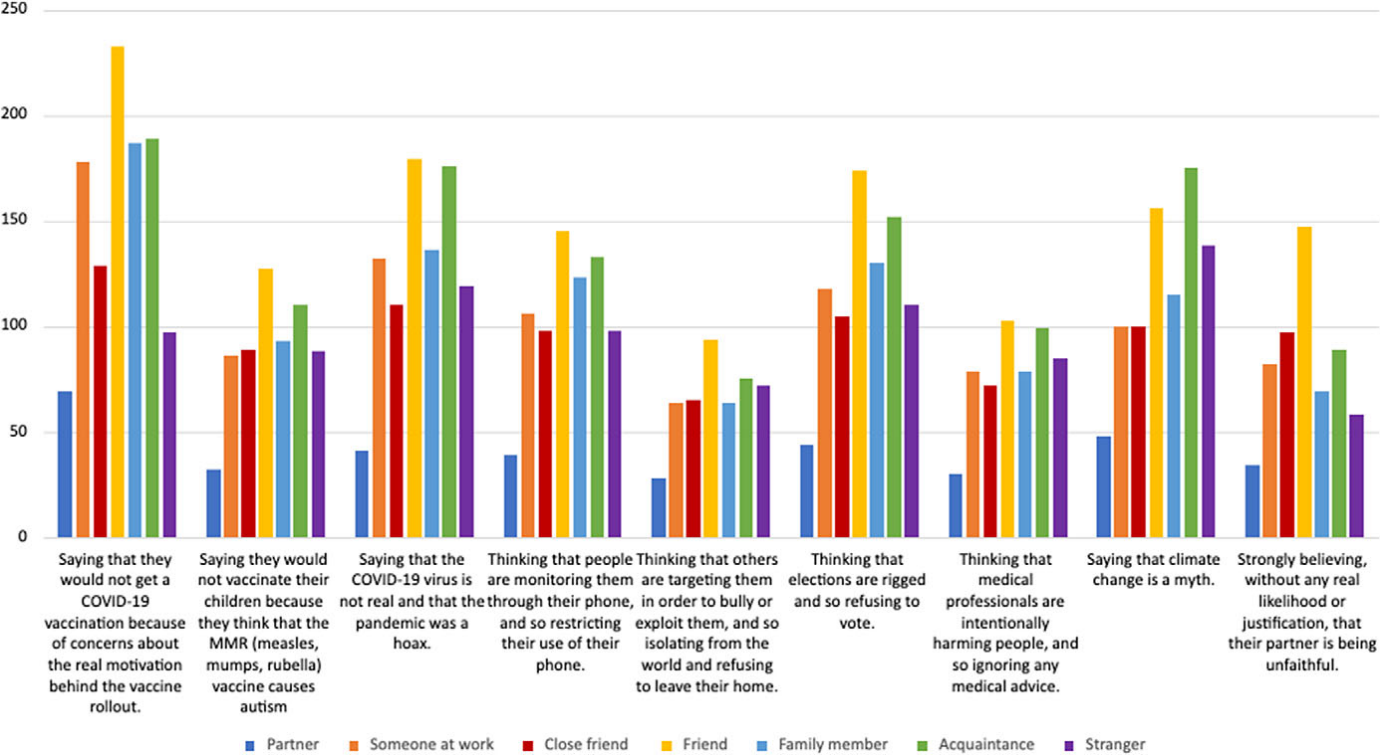
Nature of the relationship with the person who was mistrustful.

A summary of participants’ reported encounters with excessive mistrust behaviors in others is provided in Figure 1 (and see the full frequency table in Supplementary materials Table SC1). The most commonly encountered example of excessive mistrust was a person ‘Saying that they would not get a COVID-19 vaccination because of concerns about the real motivation behind the vaccine rollout,” with 698 (67.4%) participants reporting that they knew at least one person who exhibited this behavior. The least commonly encountered example was a person ‘Thinking that others are targeting them in order to bully or exploit them, and so isolating from the world and refusing to leave their home,” with 328 (31.7%) participants knowing at least one person who showed this behavior. Participants recognized excessive mistrust in other people across a range of relationships. A summary of the categories of people in which people recognized each mistrust example can be found in Figure 2 (and see the full frequency table in Supplementary materials Table SC1). On average (weighted average among those who recognized mistrust in at least one person), 155 (27.6%) participants recognized excessive mistrust in a friend, 135 (25.7%) participants in an acquaintance, 92 (17.9%) participants in a stranger, and 44 (8.6%) participants in a partner.

Out of the 1,036 respondents, 182 (17.6%) had not observed any of the nine excessive mistrust examples in other people; 85 (8.2%) participants had observed one example; 81 (7.8%) participants had observed two examples; 102 (9.8%) had observed three examples; 84 (8.1%) had observed four examples; 92 (8.9%) had observed five examples; 92 (8.9%) had observed six examples; 91 (8.8%) had observed seven examples; 81 (7.8%) had observed eight examples; and 146 (14.1%) participants had observed all nine excessive mistrust examples.

There were moderate associations between all the nine examples of observing mistrustful behavior (see Supplementary materials Table SA1; *ρ* range between .49 and .76). People were more likely to have observed mistrust behaviors if they had higher levels of paranoia themselves (R-GPTS Part A: ideas of reference subscale, *r* = 0.43, *p* < .001, N = 1036; R-GPTS Part B: ideas of persecution subscale, *r* = 0.47, *p* < 0.001, N = 1036) or higher general conspiracy belief scores (GCB score, *r* = 0.48, *p* < .001, *N* = 1036).

### Definitions of paranoia

**Figure 3.**
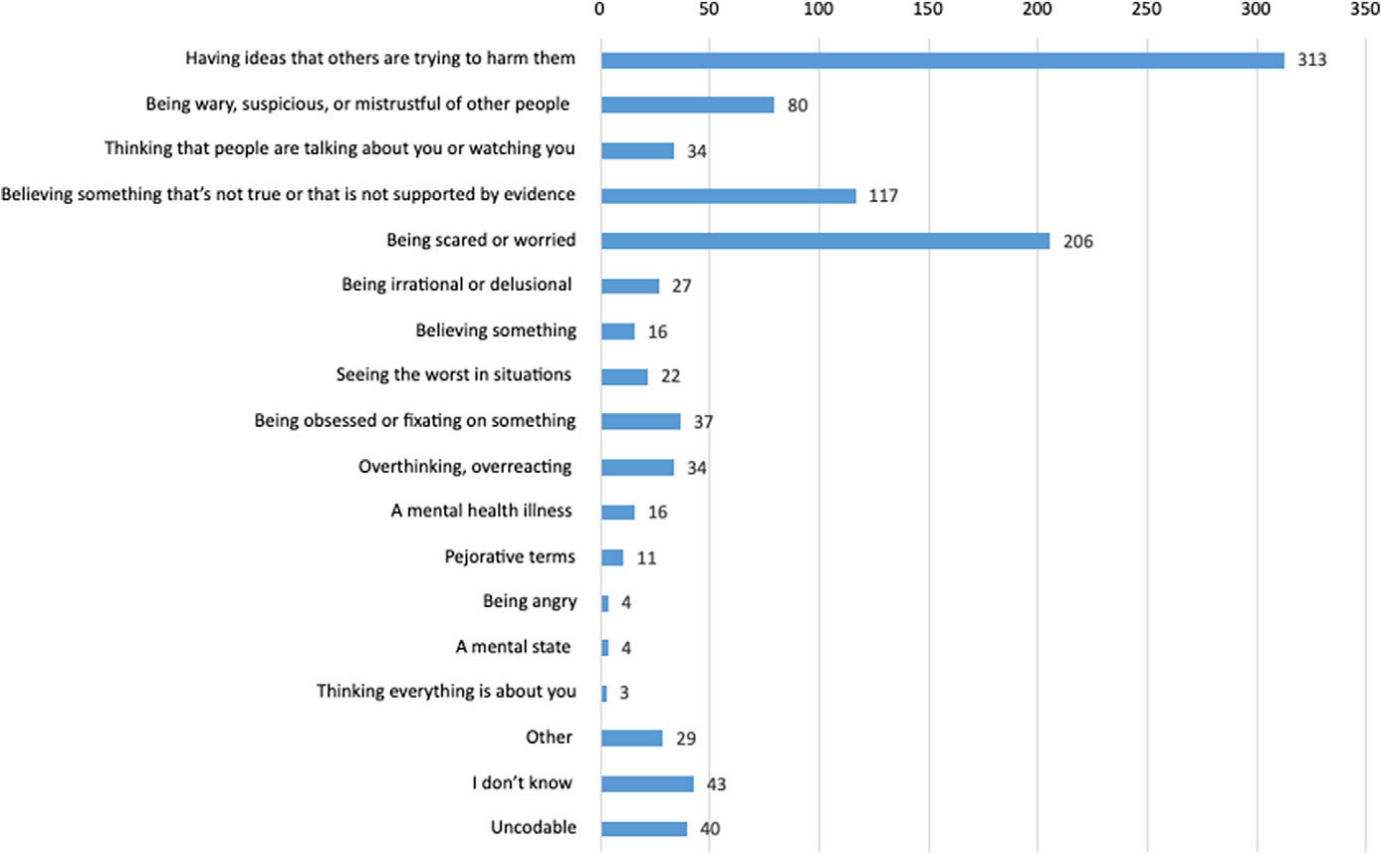
Coding of participants’ definitions of the word paranoia.

Eighteen codes were developed from the content analysis of participants’ free text definitions of paranoia. Cohen’s Kappa was calculated to assess inter-rater reliability of the coding. The Kappa value indicated an almost perfect agreement between the two raters (*κ* = 0.87). These codes are presented in Figure 3 (see Supplementary materials Table SC2 for a full frequency table with example definitions from the sample). Three hundred and thirteen (30.2%) participants defined paranoia similarly to the technical definition of paranoia (having inaccurate fears that others are trying to harm them). The next most common definition was of paranoia meaning worry or fear (*n* = 206, 19.9%).

### Experiences and understanding of paranoia

**Figure 4.**
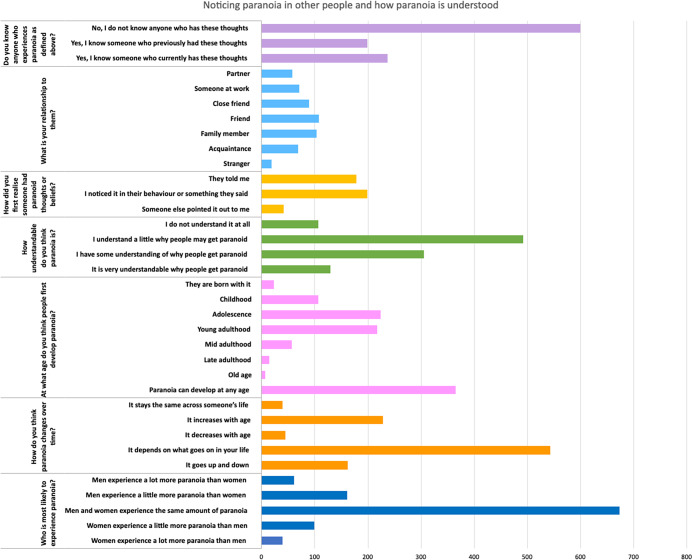
Noticing paranoia in other people and how paranoia is understood.

A summary of responses to questions about paranoia, after the provision of a definition (an enduring belief that other people (or organizations) are deliberately trying to harm you when they are not), is presented in Figure 4 (see Supplementary materials Table SC3 for a full frequency table). Four hundred and thirty-six (42.1%) participants knew someone who previously or currently experienced paranoid thoughts. Paranoia, similarly to the other examples of excessive mistrust, was also recognized in others across a range of relationships. Paranoia was least commonly recognized in strangers (*n* = 19, 4.4%).

Over half of the participant group (*n* = 600, 57.9%) reported that either they did not understand why people may get paranoid (‘I do not understand it at all’) or understood only a little (‘I understand a little why people may get paranoid’). The most endorsed opinions about the initial occurrence of paranoia were that it could either develop around ‘adolescence’ and ‘young adulthood’ (*n* = 442; 42.6%) or ‘at any age’ (*n* = 366; 35.5%). Around half of participants thought that paranoia levels increase or decrease depending on what goes on in someone’s life (‘It depends on what goes on in your life’) (*n* = 544, 52.5%). Almost two-thirds of participants (*n* = 674; 65.1%) thought that men and women experienced similar levels of paranoia.

**Figure 5.**
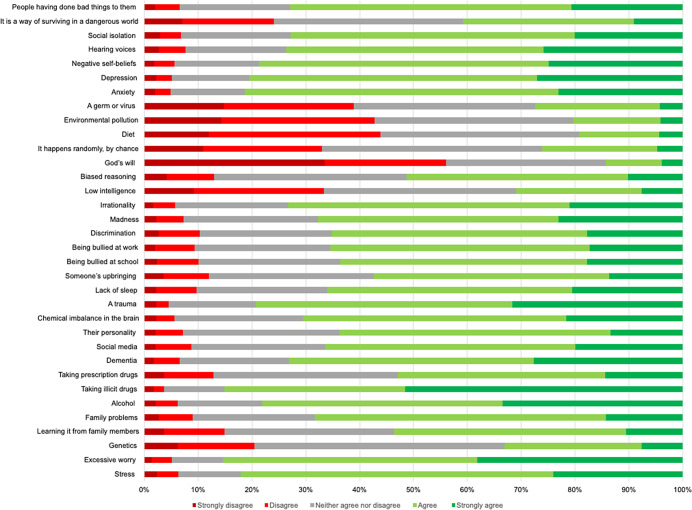
Endorsement rates for perceived causes of paranoia.

Participants endorsed a range of different causes for paranoia (see Figure 5; or Supplementary materials Table SC4 for a full frequency table). On average, participants endorsed 20 causes (*SD* = 7) out of the 34 presented. The five most ‘disagreed’ with (disagree + strongly disagree) causes for paranoia were: ‘God’s will’ (*n* = 581; 56.1%), ‘diet’ (*n* = 455; 44.0%), ‘environmental pollution’ (*n* = 444; 42.9%), ‘a germ or virus’ (*n* = 404; 39.0%), and ‘low intelligence’ (*n* = 346; 33.4%). The five potential paranoia causes that participants neither agreed nor disagreed with the most were: ‘genetics’ (*n* = 481; 46.4%), ‘chance, random’ (*n* = 424; 40.9%), ‘diet’ (*n* = 383; 37.0%), ‘environmental pollution’ (*n* = 382; 36.9%), ‘low intelligence’ (*n* = 370; 35.7%) and ‘biased reasoning’ (*n* = 370; 35.7%). The five most ‘agreed’ with (agree + strongly agree) causes for paranoia were: ‘excessive worry’ (*n* = 884; 85.3%), ‘taking illicit drugs’ (*n* = 881; 85.0%), ‘stress’ (*n* = 850; 82.0%), ‘anxiety’ (*n* = 842; 81.3%), and ‘depression’ (*n* = 834; 80.5%).

When participants were asked to indicate what they thought the main cause of paranoia was, 227 (21.9%) participants reported that ‘taking illicit drugs’ was the main cause of paranoia. 103 (9.9%) participants reported that ‘excessive worry’ was the main cause of paranoia. Ninety-seven (9.4%) reported that the main cause of paranoia was ‘stress.” Ninety-seven (9.4%) reported that the main cause of paranoia was ‘a chemical imbalance in the brain’ and 84 (8.1%) participants reported that trauma was the main cause of paranoia.

Factor analysis was used to group the responses to the potential causes of paranoia. During the EFA of the 34 causes, one cause was deleted due to low communality (<0.30) (‘taking prescription drugs’), three were deleted because they did not load onto any factor (loadings <0.30) (‘dementia,” ‘social media,” ‘lack of sleep’), and four were deleted due to cross loadings (‘someone’s upbringing,” ‘people having done bad things to them,” ‘excessive worry,” ‘stress’). With the remaining 26 causes, a seven-factor structure was identified that explained 52% of the variance: negative affect, external and uncontrollable factors, being mistreated, flawed cognition, substance use, family influences, and internal/personal vulnerabilities. Correlations between the factors were *r* = 0.10–0.46. Factor loadings and communalities for the 7-factor oblimin-rotated model derived from the EFA are presented in Table 2.

**Table 2.** Factor loadings and communalities for the oblimin rotated 7-factor structure for the causes of paranoia

Cause	Factor loading							Communality
	1	2	3	4	5	6	7	
Anxiety	0.69							0.56
Depression	0.75							0.60
Negative self-beliefs	0.66							0.58
Hearing voices	0.46							0.44
Social isolation	0.59							0.50
God’s will		0.53						0.40
It happens randomly, by chance		0.46						0.30
Diet		0.71						0.55
Environmental pollution		0.78						0.62
Germ or virus		0.71						0.55
It’s a way of surviving in a dangerous world		0.35						0.31
Being bullied at school			0.92					0.86
Being bullied at work			0.90					0.83
Discrimination			0.65					0.59
Madness				0.58				0.47
Irrationality				0.67				0.59
Low intelligence				0.39				0.33
Biased reasoning				0.53				0.37
Alcohol					0.73			0.65
Taking illicit drugs					0.66			0.62
Genetics						0.38		0.33
Learning it from family members						0.55		0.45
Family problems						0.51		0.56
Personality							0.39	0.41
Chemical imbalance in the brain							0.71	0.64
Trauma							0.35	0.46

*Note.* This table summarizes the final factor structure from the EFA (*n* = 725).

CFA in the derivation sample (*n* = 725) showed that the seven-factor model had an acceptable fit based on SRMR and RMSEA values, but the CFI and TFI suggested the model could be improved further (*χ^2^* = 1229.65, *df* = 278, *p* < 0.001, SRMR = 0.070, RMSEA = 0.069, CFI = 0.878, TLI = 0.857). CFA in the confirmatory sample (*n* = 311) demonstrated a similar model fit (*χ^2^* = 763.34, *df* = 278, *p* < 0.001, SRMR = 0.076, RMSEA = 0.075, CFI = 0.864, TLI = 0.841).

**Figure 6.**
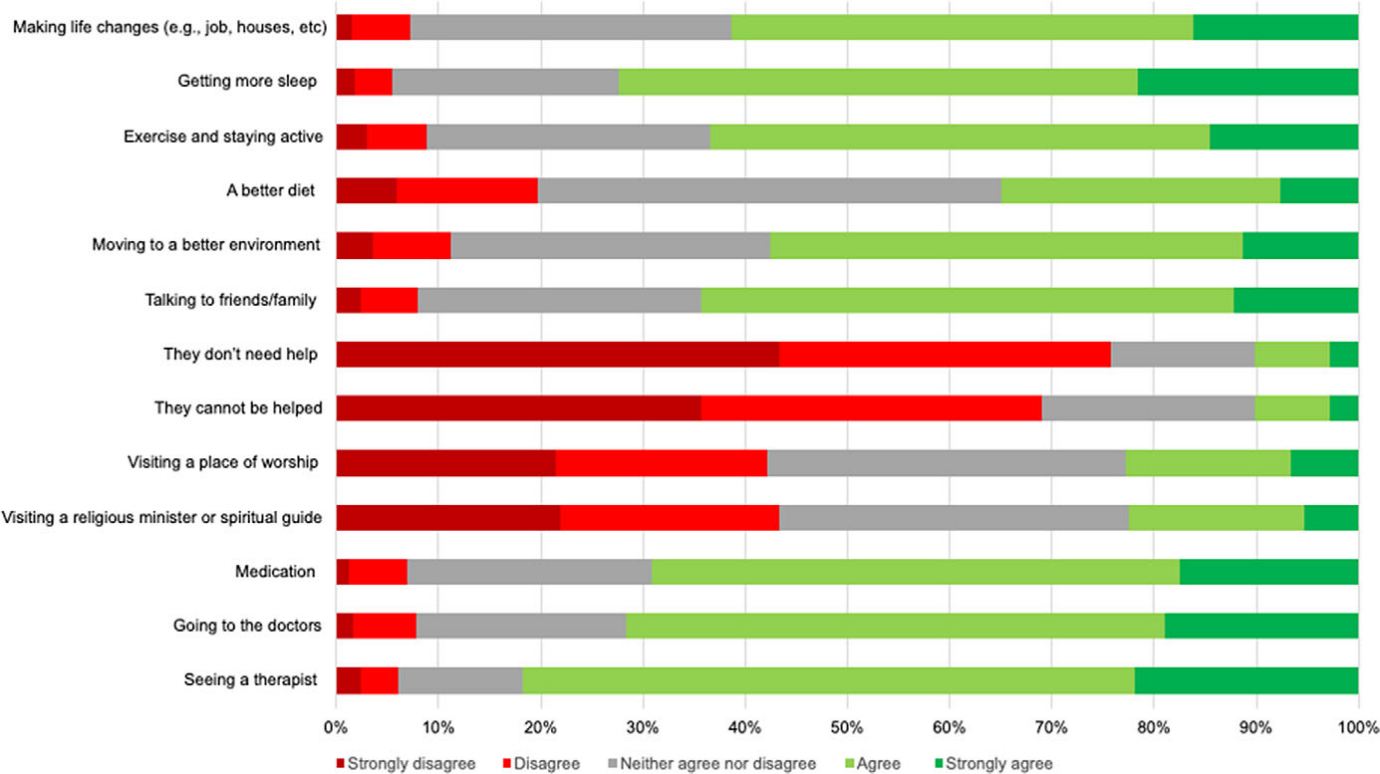
Endorsement rates for potential ways to reduce paranoia.

A summary of participants’ endorsements (or lack thereof) of potential sources of help for someone experiencing paranoia can be found in Figure 6 (see Supplementary materials Table SC4 for a full frequency table). A majority of participants neither strongly agreed nor strongly disagreed with any of the proposed factors that could help with paranoia (i.e. there was limited certainty in answers). The three most *agreed* with (agree + strongly agree) sources of help for paranoia were: ‘seeing a therapist’ (*n* = 846; 81.6%), ‘getting more sleep’ (*n* = 749; 72.3%), and ‘going to the doctors’ (*n* = 741; 71.5%). The three most *disagreed* with (disagree + strongly disagree) sources of help were: ‘they don’*t* need help’ (*n* = 785; 75.7%), ‘they cannot be helped’ (*n* = 716; 69.1%), and ‘visiting a religious minister or spiritual guide’ (*n* = 450; 43.4%). The three sources of help for paranoia that participants endorsed ‘neither agree nor disagree’ (that is may have had no view) with the most were: ‘a better diet’ (*n* = 470; 45.4%), ‘visiting a place of worship’ (*n* = 364; 35.1%), and ‘visiting a religious minister or spiritual guide’ (*n* = 354; 34.2%).

In exploratory analyses, scores for perceived causes of paranoia were compared across gender, age, ethnicity, and level of education (see Supplementary materials B: Analysis of demographic factors). A summary of mean factor scores for the total sample, as well as a breakdown by gender, age, and ethnicity can be found in Supplementary Table SB1.

Females scored significantly higher than males on sum scores for Factors 1 (negative affect), 3 (being mistreated), 5 (substance use), 6 (family influences), and 7 (internal/personal vulnerabilities), indicating greater endorsement of the corresponding items (see Supplementary Table SB2).

A MANOVA indicated that age may affect what participants endorse as causes of paranoia (*F*(28, 4112) = 3.96, *p* < 0.001). There was a significant effect of age on factor scores for the following factors (see Supplementary Table SB3): ‘external and uncontrollable factors’, ‘being mistreated’, ‘flawed cognition’, and ‘family influences’. Post-hoc Tukey’s *t*-tests revealed indicated that 25–39 year olds endorsed significantly more highly ‘external and uncontrollable factors’ and ‘family influences’ as causes for paranoia than 55–69 and 70+ year olds, and endorsed the factor ‘being mistreated’ significantly more highly than 55–69 year olds. 18–24 year olds endorsed ‘flawed cognition’ items significantly less highly than 40–54 and 70+ year olds.

A MANOVA also indicated that ethnicity may affect endorsement of causes of paranoia (*F*(28, 4068) = 2.55, *p* < 0.001). Post-hoc Tukey’s *t*-tests for Factor 2 (external and uncontrollable factors) revealed significant differences between responses for White and Asian ethnicities, with those of White ethnicity endorsing items in factor 2 (external and uncontrollable factors) significantly less often than those of Asian ethnicity.

There were no statistically significant differences in factor scores across levels of education.

## Discussion

The overwhelming majority of people who took part in the survey, representative of the general UK population on a number of basic socio-demographic factors, had encountered excessive mistrust in other people during the past year. We chose examples that were potentially observable from behavior (e.g. people talking about their fears), that were likely excessive (e.g. not taking potentially appropriate medical procedures) or false (e.g. climate change is a hoax). We asked about different domains of excessive mistrust, which were found to be moderately associated. The most commonly observed examples of excessive mistrust were related to the pandemic, which is another example of its significant impact on trust. Mistrust was primarily recognized in people the respondents knew, rather than strangers. If the respondent had higher levels of paranoia or conspiracy-type thinking, then they were more likely to report noticing such thinking in the people around them. Broadly, the results support the emerging evidence that excessive mistrust is fairly ubiquitous in the population and likely affecting behavior.

The survey had a significant focus on paranoia, given its importance in mental health. The general public’s use of the word paranoia is heterogeneous: 18 distinct categories of definitions were identified. The mental health definition of paranoia was the definition most commonly used by the participants but was still only used by just less than one-third of people. When the mental health definition of paranoia was provided to participants, the respondents were not particularly confident in their knowledge about the issue. It could well be valuable to have engaging, accessible, informational resources on the topic for the general public. However, the respondents’ views did align with the research evidence on paranoia in an important way: recognition that paranoia is mostly caused by a large number and range of factors. There were also accurate views about the emergence of paranoia in adolescence and early adulthood and that men and women experience similar levels of paranoia. Overall, participants generally disagreed with the statement that people with paranoia cannot be helped or do not need help. The importance in overcoming paranoia of both psychological therapy and medication were recognized. The significance of sleep was also highlighted, which is likely ahead of the awareness in mental health treatment provision (Freeman et al., 2017).

Our study has several limitations. First, this was a cross-sectional, anonymous, self-report study conducted online. Therefore, while we were able to check for data quality, there is no way of checking whether participants were responding honestly to the questions, and responses might be affected by a variety of self-report biases. Second, we employed a non-probability online quota sampling method, meaning that while the sample was generally representative of the UK population, the individuals may not be representative. Fourth, individuals from Asian and Asian British ethnicities were underrepresented in our study. Fifth, our assessment of participants’ recognition of excessive mistrust was limited to nine discrete examples. Although these examples captured some commonly held excessively mistrustful opinions, they are not exhaustive, and we may have overlooked other frequent or less common instances of excessive mistrust. Lastly, the survey was only conducted in one country, and results are likely to vary by location. Nevertheless, the survey provides an intriguing first snapshot of how excessive mistrust may be frequently witnessed in other people and how sense is made of it that potentially affects responses. The survey not only moves beyond the study of individual experiences of excessive mistrust, but also informs about the social world in which it is happening.

## Supporting information

Slaoui et al. supplementary material Slaoui et al. supplementary material

## Supplementary material

The supplementary material for this article can be found at http://doi.org/10.1017/S0033291725102365.
