# Supplementary material for: Are people noticing excessive mistrust in others and how do they understand it? A survey of a UK representative adult population
Source: Psychol Med. 2025 Nov 3;55:e331. doi: 10.1017/S0033291725102365 (PMC13054907; doi:10.1017/S0033291725102365)
Supplement: Slaoui et al. supplementary material [file S0033291725102365sup001.docx]

**Supplementary materials**

**Supplementary materials A: Polychoric correlations**

**Table SA1**

*Polychoric correlations between recognition scores of the nine excessive mistrust examples*

| **Variable** | **1** | **2** | **3** | **4** | **5** | **6** | **7** | **8** | **9** |
| --- | --- | --- | --- | --- | --- | --- | --- | --- | --- |
| 1. Saying that they would not get a COVID-19 vaccination because of concerns about the real motivation behind the vaccine rollout. | -- |  |  |  |  |  |  |  |  |
| 2. Saying they would not vaccinate their children because they think that the MMR (measles, mumps, rubella) vaccine causes autism | 0.65 | -- |  |  |  |  |  |  |  |
| 3. Saying that the COVID-19 virus is not real and that the pandemic was a hoax. | 0.67 | 0.64 | -- |  |  |  |  |  |  |
| 4. Thinking that people are monitoring them through their phone, and so restricting their use of their phone. | 0.61 | 0.60 | 0.62 | -- |  |  |  |  |  |
| 5. Thinking that others are targeting them in order to bully or exploit them, and so isolating from the world and refusing to leave their home. | 0.53 | 0.64 | 0.63 | 0.73 | -- |  |  |  |  |
| 6. Thinking that elections are rigged and so refusing to vote. | 0.58 | 0.61 | 0.61 | 0.64 | 0.69 | -- |  |  |  |
| 7. Thinking that medical professionals are intentionally harming people, and so ignoring any medical advice. | 0.54 | 0.63 | 0.63 | 0.66 | 0.76 | 0.71 | -- |  |  |
| 8. Saying that climate change is a myth. | 0.50 | 0.52 | 0.58 | 0.51 | 0.56 | 0.58 | 0.67 | -- |  |
| 9. Strongly believing, without any real likelihood or justification, that their partner is being unfaithful. | 0.49 | 0.58 | 0.55 | 0.60 | 0.74 | 0.62 | 0.68 | 0.53 | -- |

**Supplementary materials B: Analysis of demographic factors**

***Statistical Analyses***

To explore how paranoia is perceived and understood across different demographic groups, we compared perceived causes of paranoia across gender, age, ethnicity, and level of education.

Perceived causes of paranoia were grouped according to the seven-factor structure identified in our EFA: (1) negative affect (5 items); (2) external and uncontrollable factors (6 items); (3) being mistreated (3 items); (4) flawed cognition (4 items); (5) substance use (2 items); (6) family influences (3 items); and (7) internal/personal vulnerabilities (3 items). Each item was rated on a 5-point Likert scale (1 = Strongly disagree, 5 = Strongly agree). Factor scores were computed by summing up the items that constitute each factor. Possible score ranges were 5–25 for Factor 1 (negative affect), 6–30 for Factor 2 (external and uncontrollable factors), 3–15 for Factor 3 (being mistreated), 4-20 for Factor 4 (flawed cognition), 2-10 for Factor 5 (substance use), 3-15 for Factor 6 (family influences), and 3-15 for Factor 7 (internal/personal vulnerabilities).

To compare factor scores across genders, we conducted independent sample t-tests. As only six participants did not identify as male or female, they were excluded from analysis due to the small sample size. Consequently, factor scores were compared between male and female participants only, using Welch's t-tests, which do not assume equal variances A Bonferroni correction was applied to adjust for multiple comparisons, setting the threshold for statistical significance at *p* = .007 (i.e., .05/7).

To compare factor scores across age groups, the sample was first divided into five age categories: 18-24, 25-39, 40-54, 55-69, and 70+. To compare factor scores across ethnicities, the sample was divided into five categories: White, Asian, Black, Mixed, and Other. To compare scores across education levels, the sample was divided into six categories: No qualifications, Primary, Secondary, Further, Certificate of higher education, and Post-graduate qualifications. For each variable (age, ethnicity, and level of education), separate Multivariate Analyses of Variance (MANOVA) were conducted with age group, ethnicity, and level of education as the independent variables and the seven factor scores as the dependent variables. Following a significant MANOVA, seven one-way ANOVAs (one for each factor score) were conducted to identify where group differences occurred, setting the threshold for statistical significance at *p* = .01. Finally, post-hoc comparisons for statistically significant ANOVAs were performed using Tukey’s HSD test, again setting the threshold for statistical significance at *p* = .01.

***Results***

A summary of mean factor scores for the total sample, as well as a breakdown by gender, age, and ethnicity can be found in Table SB1.

Table SB2 summarises the results of Welch’s t-tests comparing factor scores between male and female participants. After correcting for multiple comparisons, analyses for all factors except Factor 2 (*t=-2.42, df*=1026.9, *p*=.02) and Factor 4 (*t=0.35, df*=1016.4, *p*=.73) reached statistical significance. Females scored higher than males on sum scores for Factors 1, 3, 5, 6, and 7, indicating greater endorsement of the corresponding items.

A MANOVA demonstrated that age significantly impacted what participants endorsed as causes of paranoia (*F*(28, 4112) = 3.96, *p*<.001). Table SB3 summarises the results of the one-way ANOVAs with age as the independent variable and factor scores as the dependent variables in each ANOVA. Differences in group means were statistically significant at *p*<.01 for Factors 2, 3, 4, and 6.

Post-hoc Tukey’s t-tests revealed that 25-39 year olds endorsed significantly more highly external and uncontrollable factors (Factor 2) as causes for paranoia than 55-69 (mean difference = 1.69, *95% CI* = [0.64, 2.74], *p*<.001) and 70+ year olds (mean difference = 1.89, *95% CI* = [0.50, 3.27], *p*=.002). 25-39 year olds also endorsed the factor ‘being mistreated’ (Factor 3) significantly more highly than 55-69 year olds (mean difference = 0.72, *95% CI* = [0.13, 1.30], *p*=.008). 18-24 year olds endorsed ‘flawed cognition’ (Factor 4) items significantly less highly than 40-54 (mean difference = -1.08, *95% CI* = [-2.29, -0.25], *p*=.004) and 70+ year olds (mean difference = -1.29, *95% CI* = [-2.29, -0.30], *p*=.003). Finally, post-hoc analyses revealed that 25-39 year olds endorse the ‘family influences’ factor (Factor 6) significantly more highly than 55-69 year olds (mean difference = 0.66, *95% CI* = [0.16, 1.16], *p*=.003), and 70+ year olds (mean difference = 0.79, *95% CI* = [0.13, 1.45], *p*=.009).

A MANOVA demonstrated that ethnicity significantly impacted what participants endorsed as causes of paranoia (*F*(28, 4068)=2.55, *p*<.001). One-way ANOVAs with ethnicity as the independent variable and factor scores as the dependent variables yielded statistically significant differences in group means at *p*<.01 for Factor 2 only (*F*(4, 1020)=4.23, *p*=.002).

Post-hoc Tukey’s t-tests for Factor 2 revealed significant differences between Whites and Asians (mean difference = -2.17, *95% CI* = [-3.96, -0.37], *p*=.009), with Whites endorsing items in factor 2 (external and uncontrollable factors) significantly less often than Asians.

The MANOVA showed no statistically significant differences in factor scores across levels of education *(F*(35, 5140)=1.25, *p*=.15)*.* Therefore, no post-hoc analyses were performed.

**Table SB1**

*Factor scores for the endorsement of seven categories of causes for paranoia by gender, age, and ethnicity*

| **Demographic characteristics** | | **Factor 1 negative affect** |  | **Factor 2**  **external and uncontrollable factors** |  | **Factor 3**  **being mistreated** |  | **Factor 4**  **flawed cognition** |  | **Factor 5 substance use** |  | **Factor 6**  **family influences** |  | **Factor 7 internal/**  **personal vulnerabilities** |
| --- | --- | --- | --- | --- | --- | --- | --- | --- | --- | --- | --- | --- | --- | --- |
|  |  | ***M (SD)*** |  | ***M (SD)*** |  | ***M (SD)*** |  | ***M (SD)*** |  | ***M (SD)*** |  | ***M (SD)*** |  | ***M (SD)*** |
| **Total** | | 19.65 (3.39) |  | 16.46 (4.50) |  | 11.10 (2.51) |  | 14.08 (2.70) |  | 8.34 (1.62) |  | 10.30 (2.13) |  | 11.56 (2.06) |
| **Gender** | |  |  |  |  |  |  |  |  |  |  |  |  |  |
|  | Male (n = 507) | 19.23 (3.51) |  | 16.13 (4.50) |  | 10.86 (2.62) |  | 14.11 (2.79) |  | 8.20 (1.63) |  | 10.05 (2.20) |  | 11.33 (2.15) |
|  | Female (n = 523) | 20.04 (3.24) |  | 16.80 (4.49) |  | 11.32 (2.38) |  | 14.05 (2.59) |  | 8.48 (1.61) |  | 10.54 (2.04) |  | 11.77 (1.95) |
| **Age range** | |  |  |  |  |  |  |  |  |  |  |  |  |  |
|  | 18-24 (n = 106) | 18.97 (5.11) |  | 16.41 (4.94) |  | 11.42 (2.85) |  | 13.16 (3.75) |  | 7.93 (2.00) |  | 10.38 (2.66) |  | 10.98 (2.69) |
|  | 25-39 (n = 249) | 19.56 (3.37) |  | 17.41 (4.22) |  | 11.51 (2.15) |  | 14.07 (2.46) |  | 8.24 (1.61) |  | 10.74 (1.94) |  | 11.54 (2.02) |
|  | 40-54 (n = 275) | 19.82 (3.41) |  | 16.80 (4.77) |  | 10.98 (2.69) |  | 14.24 (2.57) |  | 8.53 (1.50) |  | 10.25 (2.13) |  | 11.61 (2.12) |
|  | 55-69 (n = 294) | 19.81 (2.83) |  | 15.72 (4.21) |  | 10.79 (2.48) |  | 14.11 (2.69) |  | 8.39 (1.58) |  | 10.08 (2.05) |  | 11.63 (1.84) |
|  | 70+ (n = 112) | 19.66 (2.62) |  | 15.52 (4.28) |  | 10.97 (2.40) |  | 14.46 (2.10) |  | 8.38 (1.61) |  | 9.95 (2.06) |  | 11.85 (1.73) |
| **Ethnicity** | |  |  |  |  |  |  |  |  |  |  |  |  |  |
|  | White (n = 891) | 19.81 (3.25) |  | 16.26 (4.48) |  | 11.05 (2.51) |  | 14.05 (2.67) |  | 8.42 (1.59) |  | 10.25 (2.07) |  | 11.60 (2.03) |
|  | Asian (n = 49) | 19.20 (3.39) |  | 18.43 (4.70) |  | 11.59 (2.20) |  | 14.22 (3.04) |  | 7.82 (1.70) |  | 11.00 (2.15) |  | 11.67 (1.89) |
|  | Black (n = 44) | 19.41 (3.66) |  | 16.55 (4.38) |  | 11.77 (2.56) |  | 14.91 (2.63) |  | 8.20 (1.66) |  | 10.77 (2.61) |  | 11.34 (2.27) |
|  | Mixed (n = 36) | 18.47 (4.27) |  | 18.11 (4.25) |  | 11.22 (2.56) |  | 14.22 (2.44) |  | 7.75 (1.95) |  | 10.06 (2.24) |  | 11.31 (2.19) |
|  | Other (n = 5) | 17.80 (3.83) |  | 18.20 (2.05) |  | 11.20 (1.64) |  | 12.40 (1.82) |  | 7.80 (1.30) |  | 10.00 (2.92) |  | 10.60 (1.34) |

**Table SB2**

*Gender differences in the endorsement of seven categories of causes for paranoia*

| **Factor** | **Difference in means** | **95% CI for difference in means** | ***t*** | ***df*** | ***p*** |
| --- | --- | --- | --- | --- | --- |
| Factor 1: negative affect | -0.81 | [-1.22, -0.40] | -3.84 | 1015.3 | <0.001 |
| Factor 2: external and uncontrollable factors | -0.68 | (-1.23, -0.13] | -2.42 | 1026.9 | 0.02 |
| Factor 3: being mistreated | -0.46 | [-0.77, -0.15] | -2.95 | 1012.4 | 0.003 |
| Factor 4: flawed cognition | 0.06 | [-0.27, 0.39] | 0.35 | 1016.4 | 0.73 |
| Factor 5: substance use | -0.28 | [-0.48, -0.08] | -2.78 | 1026.1 | 0.006 |
| Factor 6: family influences | -0.49 | [-0.75, -0.23] | -3.69 | 1016.3 | <0.001 |
| Factor 7: internal/personal vulnerabilities | -0.44 | [-0.69, -0.19] | -3.45 | 1010.6 | <0.001 |

*Note*. Welch’s t-tests comparing the endorsement of each factor between male and female participants

**Table SB3**

*Age differences in the endorsement of seven categories of causes for paranoia*

| **Factor** | ***df* (between, within)** | ***F*** | ***p*** | **Partial *η²*** |
| --- | --- | --- | --- | --- |
| Factor 1: negative affect | (4, 1031) | 1.45 | .22 | .006 |
| Factor 2: external and uncontrollable factors | (4, 1031) | 6.50 | <.001 | .02 |
| Factor 3: being mistreated | (4, 1031) | 3.46 | .008 | .01 |
| Factor 4: flawed cognition | (4, 1031) | 3.93 | .004 | .02 |
| Factor 5: substance use | (4, 1031) | 2.92 | .02 | .01 |
| Factor 6: family influences | (4, 1031) | 4.34 | .002 | .02 |
| Factor 7: internal/personal vulnerabilities | (4, 1031) | 2.79 | .03 | .01 |

*Note*. One-way ANOVAs with age as the independent variable and factor scores as dependent variables.

**Supplementary materials C: Additional frequency tables**

**Table SC1**

*Observing mistrust behaviour in other people*

| **Mistrust example** | **How many people** | | | |  | **Nature of the relationship**^a^ | | | | | | |
| --- | --- | --- | --- | --- | --- | --- | --- | --- | --- | --- | --- | --- |
|  | ***n* *(%)*** | | | |  | ***n* (valid *%***^b^**)** | | | | | | |
|  | **No one** | **One or two people** | **Quite a few people** | **Most people** |  | **Partner** | **Someone at work** | **Close friend** | **Friend** | **Family member** | **Acquaintance** | **Stranger** |
| Saying that they would not get a COVID-19 vaccination because of concerns about the real motivation behind the vaccine rollout. | 338  (32.6%) | 375  (36.2%) | 259  (25.0%) | 64  (6.2%) |  | 69  (9.9%) | 178 (25.5%) | 129 (18.5%) | 233 (33.4%) | 187 (26.8%) | 189 (27.1%) | 97  (13.9%) |
| Saying they would not vaccinate their children because they think that the MMR (measles, mumps, rubella) vaccine causes autism | 570  (55.0%) | 279  (26.9%) | 160  (15.4%) | 27  (2.6%) |  | 32  (6.9%) | 86  (18.5%) | 89  (19.1%) | 127 (27.3%) | 93  (20.0%) | 110 (23.6%) | 88  (18.9%) |
| Saying that the COVID-19 virus is not real and that the pandemic was a hoax. | 433  (41.8%) | 302  (29.2%) | 228  (22.0%) | 73  (7.0%) |  | 41  (6.8%) | 132 (21.9%) | 110 (18.2%) | 179 (29.7%) | 136 (22.6%) | 176 (29.2%) | 119 (19.7%) |
| Thinking that people are monitoring them through their phone, and so restricting their use of their phone. | 527  (50.9%) | 287  (27.7%) | 168  (16.2%) | 54  (5.2%) |  | 39  (7.7%) | 106 (20.8%) | 98  (19.3%) | 145 (28.5%) | 123 (24.2%) | 133 (26.1%) | 98  (19.3%) |
| Thinking that others are targeting them in order to bully or exploit them, and so isolating from the world and refusing to leave their home. | 708  (68.3%) | 183  (17.7%) | 111  (10.7%) | 34  (3.3%) |  | 28  (8.5%) | 64  (19.5%) | 65  (19.8%) | 94  (28.7%) | 64  (19.5%) | 75  (22.9%) | 72  (22.0%) |
| Thinking that elections are rigged and so refusing to vote. | 491  (47.4%) | 277  (26.7%) | 199  (19.2%) | 69  (6.7%) |  | 44  (8.1%) | 118 (21.7%) | 105 (19.3%) | 174 (31.9%) | 130 (23.9%) | 152 (27.9%) | 110 (20.2%) |
| Thinking that medical professionals are intentionally harming people, and so ignoring any medical advice. | 656  (63.3%) | 221  (21.3%) | 122  (11.8%) | 37  (3.6%) |  | 30  (7.9%) | 79  (20.8%) | 72  (18.9%) | 103 (27.1%) | 79  (20.8%) | 99  (26.1%) | 85  (22.4%) |
| Saying that climate change is a myth. | 482  (46.5%) | 307  (29.6%) | 190  (18.3%) | 57  (5.5%) |  | 48  (8.7%) | 100 (18.1%) | 100 (18.1%) | 156 (28.2%) | 115 (20.8%) | 175 (31.6%) | 138 (24.9%) |
| Strongly believing, without any real likelihood or justification, that their partner is being unfaithful. | 619  (59.7%) | 270  (26.1%) | 109  (10.5%) | 38  (3.7%) |  | 34  (8.2%) | 82  (19.7%) | 97  (23.3%) | 147 (35.3%) | 69  (16.5%) | 89  (21.3%) | 58  (13.9%) |

^a^ Respondents could indicate more than one relationship with the person(s) being mistrustful.

^b^ Percentage of participants who indicated that they recognised the particular mistrust example in at least one or two people.

n = number of respondents.

**Table SC2**

*Participants’ definitions of the word paranoia*

| **Code** | **Example definition** | ***n*** | ***%*** |
| --- | --- | --- | --- |
| Having ideas that others are trying to harm them | “*The mistaken belief that someone or something is out to get you*” | 313 | 30.2% |
| Being wary, suspicious, or mistrustful of other people | “*I think paranoia is an unjustified suspicion and mistrust of other people or their actions*” | 80 | 7.7% |
| Thinking that people are talking about you or watching you | “*When someone feels that everyone is looking at them or talking about them and that they are being watched all the time by the government*” | 34 | 3.3% |
| Believing something that’s not true or that is not supported by evidence | “*Thinking something that’s not true or believing something that’s not real*” | 117 | 11.3% |
| Being scared or worried | “*Anxious and worried about something, scared its gonna happen*” | 206 | 19.9% |
| Being irrational or delusional | “*Irrational beliefs about people or situations*” | 27 | 2.6% |
| Believing something | “*State of mind where someone believes something in a way that no one can persuade them otherwise*” | 16 | 1.5% |
| Seeing the worst in situations | “*Thinking everything is wrong all the time*” | 22 | 2.1% |
| Being obsessed or fixating on something | “*Someone who is overly obsessed with something*” | 37 | 3.6% |
| Overthinking, overreacting | “*Overthinking certain situations to an extreme level*” | 34 | 3.3% |
| A mental health illness | “*Paranoia is a mental illness*” | 16 | 1.5% |
| Pejorative terms | “*Weird*” | 11 | 1.1% |
| Being angry | “*Paranoia is a form of anger traits in a person*” | 4 | 0.4% |
| A mental state | “*Paranoia is a term used to describe a mental state*” | 4 | 0.4% |
| Thinking everything is about you | “*People think every thing is about them*” | 3 | 0.3% |
| Other | “*A made up term to attempt to contradict the masses*” | 29 | 2.8% |
| I don’t know | “*I don’t know what that means*” | 43 | 4.2% |
| Uncodable | “*Feeling paranoid about something*” | 40 | 3.9% |

**Table SC3**

*Noticing paranoia in other people and how paranoia is understood*

| **Question** | **Response** | ***n*** | ***%*** |
| --- | --- | --- | --- |
| **Do you know anyone who experiences paranoia as defined above?** |  |  |  |
|  | No, I do not know anyone who has these thoughts | 600 | 57.9% |
|  | Yes, I know someone who previously had these thoughts | 199 | 19.2% |
|  | Yes, I know someone who currently has these thoughts | 237 | 22.9% |
| **What is your relationship to them?** ^a^ |  |  |  |
|  | Partner | 58 | 13.3% |
|  | Someone at work | 71 | 16.3% |
|  | Close friend | 90 | 20.6% |
|  | Friend | 108 | 24.8% |
|  | Family member | 104 | 23.9% |
|  | Acquaintance | 69 | 15.8% |
|  | Stranger | 19 | 4.4% |
| **How did you first realise someone had paranoid thoughts or beliefs?** ^a^ |  |  |  |
|  | They told me | 179 | 41.1% |
|  | I noticed it in their behaviour or something they said | 199 | 45.6% |
|  | Someone else pointed it out to me | 42 | 9.6% |
| **How understandable do you think paranoia is?** |  |  |  |
|  | I do not understand it at all | 107 | 10.3% |
|  | I understand a little why people may get paranoid | 493 | 47.6% |
|  | I have some understanding of why people get paranoid | 306 | 29.5% |
|  | It is very understandable why people get paranoid | 130 | 12.5% |
| **At what age do you think people first develop paranoia?** |  |  |  |
|  | They are born with it | 23 | 2.2% |
|  | Childhood | 107 | 10.3% |
|  | Adolescence | 224 | 21.6% |
|  | Young adulthood | 218 | 21.0% |
|  | Mid adulthood | 57 | 5.5% |
|  | Late adulthood | 15 | 1.4% |
|  | Old age | 7 | 0.7% |
|  | Paranoia can develop at any age | 366 | 35.3% |
| **How do you think paranoia changes over time?** |  |  |  |
|  | It stays the same across someone’s life | 40 | 3.9% |
|  | It increases with age | 229 | 22.1% |
|  | It decreases with age | 45 | 4.3% |
|  | It depends on what goes on in your life | 544 | 52.5% |
|  | It goes up and down | 163 | 15.7% |
| **Who is most likely to experience paranoia?** |  |  |  |
|  | Men experience a lot more paranoia than women | 62 | 6.0% |
|  | Men experience a little more paranoia than women | 161 | 15.5% |
|  | Men and women experience the same amount of paranoia | 674 | 65.1% |
|  | Women experience a little more paranoia than men | 99 | 9.6% |
|  | Women experience a lot more paranoia than men | 40 | 3.9% |

^a^ These questions were only answered by respondents who indicated that they knew someone who currently or previously held paranoid ideas (n = 436). The percentage was calculated with regards to the adjusted sample size.

n = number of respondents.

**Table SC4**

*Perceived causes of paranoia*

| **Factor** | **Strongly disagree** | |  | **Disagree** | |  | **Neither agree nor disagree** | |  | **Agree** | |  | **Strongly agree** | |
| --- | --- | --- | --- | --- | --- | --- | --- | --- | --- | --- | --- | --- | --- | --- |
|  | ***n*** | ***%*** |  | ***n*** | ***%*** |  | ***n*** | ***%*** |  | ***n*** | ***%*** |  | ***n*** | ***%*** |
| Stress | 25 | 2.4% |  | 41 | 4.0% |  | 120 | 11.6% |  | 602 | 58.1% |  | 248 | 23.9% |
| Excessive worry | 15 | 1.4% |  | 38 | 3.7% |  | 99 | 9.6% |  | 489 | 47.2% |  | 395 | 38.1% |
| Genetics | 65 | 6.3% |  | 148 | 14.3% |  | 481 | 46.4% |  | 263 | 25.4% |  | 79 | 7.6% |
| Learning it from family members | 38 | 3.7% |  | 117 | 11.3% |  | 326 | 31.5% |  | 447 | 43.1% |  | 108 | 10.4% |
| Family problems | 29 | 2.8% |  | 64 | 6.2% |  | 236 | 22.8% |  | 560 | 54.1% |  | 147 | 14.2% |
| Alcohol | 22 | 2.1% |  | 43 | 4.2% |  | 162 | 15.6% |  | 463 | 44.7% |  | 346 | 33.4% |
| Taking illicit drugs | 18 | 1.7% |  | 21 | 2.0% |  | 116 | 11.2% |  | 348 | 33.6% |  | 533 | 51.4% |
| Taking prescription drugs | 39 | 3.8% |  | 94 | 9.1% |  | 356 | 34.4% |  | 398 | 38.4% |  | 149 | 14.4% |
| Dementia | 18 | 1.7% |  | 51 | 4.9% |  | 210 | 20.3% |  | 471 | 45.5% |  | 286 | 27.6% |
| Social media | 22 | 2.1% |  | 69 | 6.7% |  | 258 | 24.9% |  | 481 | 46.4% |  | 206 | 19.9% |
| Their personality | 22 | 2.1% |  | 53 | 5.1% |  | 301 | 29.1% |  | 521 | 50.3% |  | 139 | 13.4% |
| Chemical imbalance in the brain | 23 | 2.2% |  | 36 | 3.5% |  | 247 | 23.8% |  | 507 | 48.9% |  | 223 | 21.5% |
| A trauma | 23 | 2.2% |  | 24 | 2.3% |  | 168 | 16.2% |  | 494 | 47.7% |  | 327 | 31.6% |
| Lack of sleep | 23 | 2.2% |  | 78 | 7.5% |  | 251 | 24.2% |  | 472 | 45.6% |  | 212 | 20.5% |
| Someone’s upbringing | 37 | 3.6% |  | 88 | 8.5% |  | 317 | 30.6% |  | 453 | 43.7% |  | 141 | 13.6% |
| Being bullied at school | 25 | 2.4% |  | 80 | 7.7% |  | 272 | 26.3% |  | 475 | 45.8% |  | 184 | 17.8% |
| Being bullied at work | 21 | 2.0% |  | 76 | 7.3% |  | 262 | 25.3% |  | 498 | 48.1% |  | 179 | 17.3% |
| Discrimination | 29 | 2.8% |  | 78 | 7.5% |  | 254 | 24.5% |  | 492 | 47.5% |  | 183 | 17.7% |
| Madness | 24 | 2.3% |  | 52 | 5.0% |  | 259 | 25.0% |  | 463 | 44.7% |  | 238 | 23.0% |
| Irrationality | 17 | 1.6% |  | 43 | 4.2% |  | 218 | 21.0% |  | 541 | 52.2% |  | 217 | 20.9% |
| Low intelligence | 96 | 9.3% |  | 250 | 24.1% |  | 370 | 35.7% |  | 242 | 23.4% |  | 78 | 7.5% |
| Biased reasoning | 43 | 4.2% |  | 92 | 8.9% |  | 370 | 35.7% |  | 426 | 41.1% |  | 105 | 10.1% |
| God’s will | 348 | 33.6% |  | 233 | 22.5% |  | 308 | 29.7% |  | 108 | 10.4% |  | 39 | 3.8% |
| It happens randomly, by chance | 113 | 10.9% |  | 229 | 22.1% |  | 424 | 40.9% |  | 221 | 21.3% |  | 49 | 4.7% |
| Diet | 125 | 12.1% |  | 330 | 31.9% |  | 383 | 37.0% |  | 153 | 14.8% |  | 45 | 4.3% |
| Environmental pollution | 149 | 14.4% |  | 295 | 28.5% |  | 382 | 36.9% |  | 168 | 16.2% |  | 42 | 4.1% |
| A germ or virus | 154 | 14.9% |  | 250 | 24.1% |  | 348 | 33.6% |  | 241 | 23.3% |  | 43 | 4.2% |
| Anxiety | 21 | 2.0% |  | 30 | 2.9% |  | 143 | 13.8% |  | 604 | 58.3% |  | 238 | 23.0% |
| Depression | 24 | 2.3% |  | 29 | 2.8% |  | 149 | 14.4% |  | 554 | 53.5% |  | 280 | 27.0% |
| Negative self-beliefs | 20 | 1.9% |  | 38 | 3.7% |  | 163 | 15.7% |  | 558 | 53.9% |  | 257 | 24.8% |
| Hearing voices | 28 | 2.7% |  | 52 | 5.0% |  | 194 | 18.7% |  | 495 | 47.8% |  | 267 | 25.8% |
| Social isolation | 31 | 3.0% |  | 40 | 3.9% |  | 212 | 20.5% |  | 546 | 52.7% |  | 207 | 20.0% |
| It is a way of surviving in a dangerous world | 74 | 7.1% |  | 176 | 17.0% |  | 364 | 35.1% |  | 329 | 31.8% |  | 93 | 9.0% |
| People having done bad things to them | 21 | 2.0% |  | 48 | 4.6% |  | 212 | 20.5% |  | 542 | 52.3% |  | 213 | 20.6% |

**Table SC5**

*Endorsement rates for potential ways to reduce paranoia*

| **Factor** | **Strongly disagree** | |  | **Disagree** | |  | **Neither agree nor disagree** | |  | **Agree** | |  | **Strongly agree** | |
| --- | --- | --- | --- | --- | --- | --- | --- | --- | --- | --- | --- | --- | --- | --- |
|  | ***n*** | ***%*** |  | ***n*** | ***%*** |  | ***n*** | ***%*** |  | ***n*** | ***%*** |  | ***n*** | ***%*** |
| Seeing a therapist | 25 | 2.4% |  | 39 | 3.8% |  | 126 | 12.2% |  | 620 | 59.8% |  | 226 | 21.8% |
| Going to the doctors | 18 | 1.7% |  | 64 | 6.2% |  | 213 | 20.6% |  | 545 | 52.6% |  | 196 | 18.9% |
| Medication | 13 | 1.3% |  | 59 | 5.7% |  | 248 | 23.9% |  | 535 | 51.6% |  | 181 | 17.5% |
| Visiting a religious minister or spiritual guide | 227 | 21.9% |  | 223 | 21.5% |  | 354 | 34.2% |  | 178 | 17.2% |  | 54 | 5.2% |
| Visiting a place of worship | 223 | 21.5% |  | 214 | 20.7% |  | 364 | 35.1% |  | 167 | 16.1% |  | 68 | 6.6% |
| They cannot be helped | 371 | 35.8% |  | 345 | 33.3% |  | 216 | 20.8% |  | 75 | 7.2% |  | 29 | 2.8% |
| They don’t need help | 450 | 43.4% |  | 335 | 32.3% |  | 146 | 14.1% |  | 76 | 7.3% |  | 29 | 2.8% |
| Talking to friends/family | 25 | 2.4% |  | 58 | 5.6% |  | 287 | 27.7% |  | 540 | 52.1% |  | 126 | 12.2% |
| Moving to a better environment | 37 | 3.6% |  | 80 | 7.7% |  | 324 | 31.3% |  | 479 | 46.2% |  | 116 | 11.2% |
| A better diet | 62 | 6.0% |  | 143 | 13.8% |  | 470 | 45.4% |  | 283 | 27.3% |  | 78 | 7.5% |
| Exercise and staying active | 31 | 3.0% |  | 61 | 5.9% |  | 288 | 27.8% |  | 506 | 48.8% |  | 150 | 14.5% |
| Getting more sleep | 20 | 1.9% |  | 37 | 3.6% |  | 230 | 22.2% |  | 526 | 50.8% |  | 223 | 21.5% |
| Making life changes (e.g., job, houses, etc) | 16 | 1.5% |  | 59 | 5.7% |  | 326 | 31.5% |  | 468 | 45.2% |  | 167 | 16.1% |
